# Co-Operation Once More

**Published:** 1913-04-12

**Authors:** A. P. Hughes Gibb

**Affiliations:** Dreadnought, Greenwich


### Co-operation Once More.

*To the Editor of* The Hospital.

Sir,—It is somewhat surprising to find the apparent agreement on all sides with the suggestions for close co-operation between the hospitals and the infirmaries which have recently appeared in The Hospital. But it is to be feared that this agreement is more apparent than real. There seems to be no question that Poor-Law infirmaries should be removed from the control of individual Boards of Guardians and placed under the authority of the Metropolitan Asylums Board, but as to what they will then become and their relation to the voluntary hospital there seems considerable doubt.

It will only be possible to carry through any large scheme of this kind with the consent of the ratepayers as a whole and the support of the Local Government Board. It seems important, therefore, to remember what their attitude is likely to be. It may probably be summed up in one word, "economy." No scheme involving large additional permanent expenditure has much chance of being sanctioned. Now it is perfectly natural that a keen and able infirmary superintendent should desire to see his institution placed on an equal footing with the best voluntary hospitals as regards staff, equipment, and treatment; while even an independent authority—Sir Henry Burdett—has written in favour of the infirmaries being thrown open to clinical teaching. If such a course were adopted it would be the death blow of the voluntary hospital. No general hospital with a medical school could long withstand the competition of a large rate-supported institution in its immediate neighbourhood with all the resources of the rates and the State with which to meet the needs of medical education and the advances of medical science. The surgeon or physician is a specialist and in many cases no layman can judge of the real importance or permanent value of the changes which he demands, often quite rightly, in the buildings or organisation of a voluntary hospital. But lack of funds, the immense difficulty of finding the money, acts as a salutary check on extravagant requests, and only those needs which are really urgent and necessary are pressed. Thus the balance between economy and efficiency is fairly well kept. In the case of a rate-supported institution no such check would be in existence; everyone knows that the money can be had if a good enough case is made out for its expenditure. Even the permanent official is wax in the hands of the specialist. Under such conditions staff, students, and cases would be attracted from the voluntary to the State-supported hospital and the former would sink below the position now occupied by the infirmaries. Whatever the arguments in favour of such a result, it is clear that neither the ratepayer nor the Local Government Board would support a scheme involving such great additional responsibility and expense.

Thus the whole scheme of co-operation between the hospital and the infirmary, carrying with it increased opportunities of clinical teaching and improved treatment for the destitute poor, would break down because it would have been made to serve the purpose of a mistaken ideal. The only hope for co-operation is in the maintenance of the present position of hospital and infirmary; the one for specialist and acute treatment, the other for simple and chronic ailments. While there are many difficulties of administration in the way of a system of transferring cases from hospital to infirmary and *vice versa*, they do not appear to be insuperable. At any rate such a change would strengthen rather than weaken the voluntary hospital system and would give to the infirmaries just the work which they are best qualified to do.—Yours, etc.,

A. P. Hughes Gibb.

Dreadnought, Greenwich, April 7, 1913.

[We welcome Mr. Hughes Gibb's letter because we wish the points made in the articles of our Special Number of last week to be the subject of discussion. But we postpone our reply to criticisms in order to give both hospital and infirmary authorities of all grades an opportunity of expressing their views on co-operation.—Ed. The Hospital.]

